# De-implementing public health policies: a qualitative study of the process of implementing and then removing body mass index (BMI) report cards in Massachusetts public schools

**DOI:** 10.1186/s43058-023-00443-1

**Published:** 2023-06-09

**Authors:** Mary Kathryn Poole, Rebekka M. Lee, Kelsey L. Kinderknecht, Erica L. Kenney

**Affiliations:** 1 Department of Nutrition, Harvard T.H. Chan School of Public Health, 665 Huntington Avenue, Boston, MA 02115 USA; 2 Department of Social and Behavioral Sciences, Harvard T.H. Chan School of Public Health, 677 Huntington Avenue, Boston, MA 02115 USA

**Keywords:** De-implementation, Implementation science, Public health, Public policy

## Abstract

**Background:**

This study explored reasons for the adoption of a policy to distribute report cards to parents about children’s weight status (“BMI report cards”) in Massachusetts (MA) public schools in 2009 and the contextual factors influencing the policy removal in 2013.

**Methods:**

We conducted semi-structured, qualitative interviews with 15 key decision-makers and practitioners involved with implementing and de-implementing the MA BMI report card policy. We analyzed interview data using a thematic analytic approach guided by the Consolidated Framework for Implementation Research (CFIR) 2.0.

**Results:**

Primary themes were that (1) factors other than scientific evidence mattered more for policy adoption, (2) societal pressure spurred policy adoption, (3) problems with the policy design contributed to inconsistent implementation and dissatisfaction, and (4) media coverage, societal pressure, and organizational politics and pressure largely prompted de-implementation.

**Conclusions:**

Numerous factors contributed to the de-implementation of the policy. An orderly process for the de-implementation of a policy in public health practice that manages drivers of de-implementation may not yet exist. Public health research should further focus on how to de-implement policy interventions when evidence is lacking or there is potential for harm.

**Supplementary Information:**

The online version contains supplementary material available at 10.1186/s43058-023-00443-1.

Contributions to the literature

- This study helps to fill a gap in de-implementation literature by describing how and why a state-level health policy was de-implemented in public health practice as well as the perceptions of de-implementation among individuals with a variety of roles involved with policy development, administration, and implementation.
- The criteria for evaluating whether an intervention is a candidate for de-implementation, such as being ineffective, costly, or harmful, are not the only reasons public health policies are removed from practice.
- De-implementation strategies and processes should be informed by the contextual factors driving de-implementation which may differ from those for intervention adoption and implementation.

## Background

The implementation of evidence-based policy interventions is essential to advancing population health, but an equally important consideration is how to *de-*implement policies that do not work or have harmful consequences. De-implementation, a relatively new concept with emerging theoretical constructs and methods in the implementation science field, involves the discontinuation or removal of interventions from practice [1]. McKay and colleagues proposed several criteria for de-implementation, including (1) if an intervention is ineffective or harmful, (2) if an alternative intervention is identified that makes better use of resources or is more effective, or (3) if the targeted health issue is no longer a priority [1]. The use of these criteria for considering de-implementation may be particularly useful to policymakers and practitioners in public health practice, where available funds for public health initiatives are often minimal [2], in order to maximize impacts on health outcomes.

Gaps in evidence for de-implementation remain for several key areas. First, it has been suggested that factors influencing policy adoption may not be the same as those for de-implementation [3]; however, examples of these differences are limited. Another knowledge gap is what the de-implementation process looks like for public health policies since the de-implementation literature has focused primarily on low-value care in clinical settings [4-7]. Evidence is also limited for how perspectives of de-implementation compare and contrast [3] by the roles of the individuals involved with the policy implementation.

Body mass index (BMI) report cards, state-level policies requiring schools to distribute “report cards” of weight status to parents/guardians (distinct from state policies that require schools to collect heights and weights for surveillance purposes, but involve anonymous data collection and no report-backs to parents), have been cited as a potential candidate for de-implementation [8-10]. BMI report cards were initially proposed in the early 2000s as a strategy for childhood obesity prevention [11]. Eleven states adopted this policy in the past 20 years, whereas another 14 states implemented screening-only policies for public health surveillance [12, 13]. Initially, there was little evidence for the impacts of BMI report cards on their intended purpose of reducing childhood obesity with several earlier reviews noting that existing evidence for an impact on behaviors was mixed [14] and that there was little evidence for an impact on obesity prevalence [12, 15]. However, as time has passed, and this policy has been able to be further evaluated, evidence from a well-designed randomized controlled trial in 2021 [9] and natural experiment in 2011 [16] has shown BMI report cards do not prevent or reduce childhood obesity. Additionally, evidence indicates BMI report cards have received negative feedback from parents [17, 18] and may increase weight dissatisfaction among participating children [9] which is a risk factor for the use of unhealthy weight control behaviors [19, 20].

Our study used qualitative interviews, guided by the Consolidated Framework for Implementation Research (CFIR 2.0) [21], to understand the process by which policymakers, practitioners, and community advisors adopted and then de-implemented a BMI report card policy in Massachusetts (MA). We had three study aims: (1) to explore the reasons for policy adoption, (2) to identify the contextual factors that influenced removing the policy from practice, and (3) to understand the acceptability and feasibility of de-implementation of the policy and the de-implementation process [3].

## Methods

### Study design and sample

**Fig. 1:**
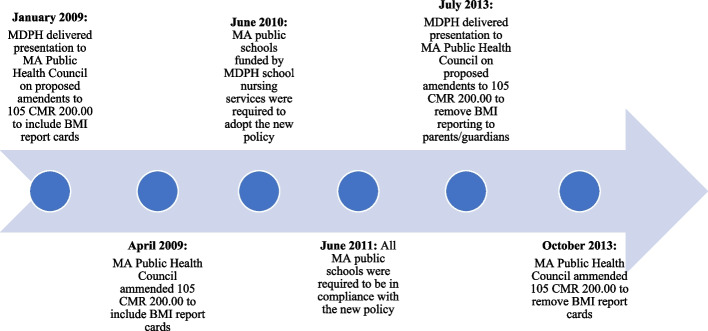
Timeline of MA BMI report card policy adoption, implementation, and de-implementation. MA, Massachusetts; MDPH, Massachusetts Department of Public Health

This qualitative study employed semi-structured interviews of individuals involved with the MA BMI report card policy implementation between April 2009 and October 2013 and/or policy de-implementation in October 2013 (see Fig. 1).

In 2009, the MA Public Health Council amended regulations governing physical examination of students (105 CMR 200.00) to include a provision for BMI report cards [13]. The “adoption” of the BMI report card policy involved a policy amendment to eliminate the existing requirement for annual height and weight surveillance in public schools and instead required BMI screening and reporting for students in grades 1, 4, 7, and 10. BMI *screening* refers to two components: (1) the assessment of student height, weight, and BMI at school and (2) the reporting of aggregate BMI results to the MA Department of Public Health (MDPH) to track obesity prevalence. BMI *reporting* refers to the requirement for BMI results to be delivered to parents/guardians as a “report card” along with nutrition and physical activity resources. De-implementation of the BMI report cards occurred in October 2013 when the MA Public Health Council amended the regulations (105 CMR 200.00) to exclude BMI report cards for parents/guardians; however, the council preserved provisions for BMI screening and aggregate reporting of results to MDPH [13].

We purposively sampled participants representing major roles of those involved with the implementation and de-implementation of the BMI report card policy, including (1) members of the MA Public Health Council which oversees state public health policies [22]; (2) leadership from MDPH, the entity responsible for policy implementation; (3) representatives from partner agencies involved with program development; and (4) school nurses responsible for program delivery. In February 2022, we reviewed historical documents, including MDPH program manuals and MA Public Health Council meeting minutes, to generate a sampling frame of potential participants (*n* = 43). Additional suggestions for individuals to recruit were obtained from study participants (*n* = 7). We also created a timeline of key events from our review of the historical documents (see Fig. 1) and verified it for accuracy with key personnel involved with the policy implementation and de-implementation.

From April to July 2022, we recruited participants by email or list serv and offered a $25 gift card incentive. We prioritized and contacted 21 individuals based on their roles and level of involvement with implementation and de-implementation. Of these, 2 declined and 4 did not respond after 3 communication attempts. After recruiting and conducting 15 interviews with representation from key stakeholder groups, we reached saturation of themes by having adequate information power [23]  to accomplish our study aims and sample specificity. This study was determined exempt by the Institutional Review Board at the Harvard T.H. Chan School of Public Health. Data were reported according to the Standards for Reporting Qualitative Research checklist (see Additional file 1).

### Interviews

We conducted semi-structured interviews using interview questions guided by CFIR 2.0 [21] and by interview questions from our team’s prior work [24] informed by the original CFIR [25] and publicly available CFIR questions [26]. The CFIR 2.0 features constructs that are associated with effective implementation across five domains: (1) the Innovation, meaning the intervention or service being implemented; (2) the Inner Setting where the intervention is implemented; (3) the Outer Setting where the Inner Setting is situated; (4) the Individuals involved and how they relate to implementation; and (5) the Implementation Process [21]. Three study authors (RL, EK, MKP) collaborated on the development of the interview questions and protocols. The open-ended interview questions asked about the participant’s role and key determinants for implementation [21, 25], including the factors influencing policy implementation and de-implementation, perceptions of the policy, impact, and feasibility of steps for implementation and de-implementation (see Additional file 2 for interview questions).

Based on our verified timeline of historical events, we included our definitions of the timing for “implementation” (April 2009 to October 2013) and “de-implementation” (October 2013) in the study recruitment materials, consent script, and interview questions to help remind participants of the timeline. We also organized our interview questions by these periods of time to distinguish between data for implementation and de-implementation.

All study authors conducted and audio-recorded the 45- to 60-min interviews using Zoom. We used Sonix software [27] to transcribe interview audio files to text and checked the transcripts for accuracy.

### Data analysis

**Fig. 2:**
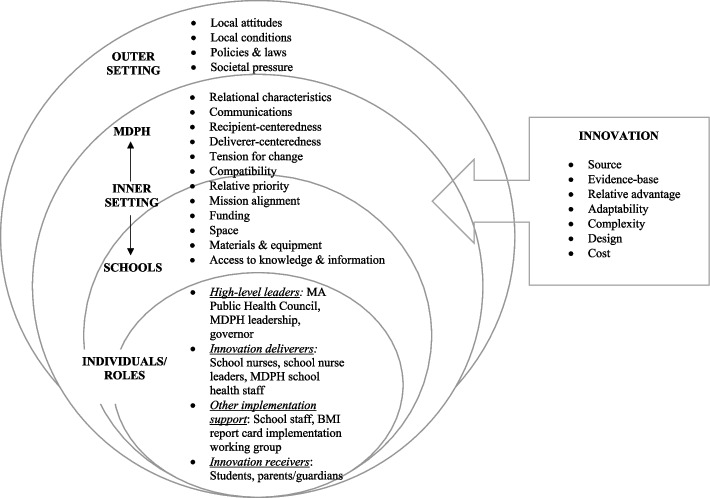
Conceptual model of CFIR 2.0 constructs for MA BMI report card policy implementation and de-implementation. MDPH, Massachusetts Department of Public Health

We imported transcripts into NViVo qualitative software [28] to conduct a thematic analysis informed by CFIR 2.0 to characterize factors influencing policy implementation and de-implementation [21]. Two study authors (MKP, KK) used a deductive approach to develop a codebook of CFIR constructs across four CFIR 2.0 domains: Innovation, Inner Setting, Outer Setting, and Individuals/Roles subdomain (Fig. 2). The two study authors independently coded the same transcripts from two interviews using a line by line analysis. They also reviewed the transcripts to determine if any new inductive codes beyond the CFIR framework were needed. Next, they compared coded text, reached consensus on codes, and adjusted the codebook following discussions with all study authors. Codebook revisions included the omission of repetitive codes and the clarification of when certain codes should be applied to text. No codes were added. The two study authors used the revised codebook to modify their coding of the same two transcripts, and then they double coded three additional transcripts (26%). The process of comparing codes, reaching consensus, and meeting with all study authors was repeated. The two study authors then divided and independently coded the remaining 11 interviews using the revised codebook and met to discuss areas of uncertainty before finalizing the coded transcripts and summarizing the coded data by CFIR domains. All study authors then independently reviewed the summarized codes and text to identify preliminary themes within and across CFIR domains. The team met to share and refine preliminary themes, discuss additional themes, and reach consensus on themes.

Throughout the process of conducting interviews, developing the codebook, and analyzing and interpreting the data, the authors (MKP, RL, KK, EK) acknowledged how their life experiences and identities related to the data. MKP, a former 4-year MA resident, is a researcher and prior public health practitioner with experience implementing school-based childhood obesity prevention initiatives, including in a state with BMI report cards. RL, a lifelong MA resident, conducts research on childhood obesity prevention, weight stigma, and implementation science and has partnerships with MA government agencies. KK, a 2-year MA resident, is a researcher and former public health practitioner with experience implementing school-based nutrition initiatives. EK, a long-time MA resident whose family members have attended MA public schools across three generations conducts research on childhood obesity prevention and weight stigma.

## Results

Of the* n* = 15 interview participants, five were current or former staff at MDPH; one was a former member of the MA Public Health Council; three were school nurses; and six were community advisors/practitioners. Identified themes are organized by our original study aims below; examples of representative quotations are provided with the associated CFIR 2.0 domains and constructs listed in parenthesis. Additional quotations are displayed in Table 1.

**Table 1:** Themes and examples of factors influencing implementation and de-implementation of the MA BMI report card policy

Theme and corresponding CFIR 2.0 domain and construct	Illustrative quotations
**Aim 1: To explore the reasons for adoption of the BMI report card policy**	
**Theme:** The evidence base was not the primary motivator for adoption—instead, the fact that the BMI report cards came from trusted sources mattered more Innovation ⇒* Evidence Base* *⇒ Source*	*Role: Other implementation support—Community advisor:* “And some of it was, you know, some of the implementation and the sense that this was the right thing to do and that we knew how to do. It was based on the Cambridge Public Schools having done this for several years.”
	*Role: High-level leader—MDPH:* “Now, when you're a strong leader and most of what you do is really good, you can easily get people to just say, not question you, right? To say, Oh, yeah, [she’s] smart, she's good, she's done all these great things. So this is just another one of those things. So we'll go forth…I don't think that they had enough feedback to ask any kind of questions about it. So it passed with flying colors and then as soon as it got implemented, people started going crazy.”
	*Role: Other implementation support—Community advisor: “*But the state was always very clear, as was Cambridge, that surveillance was really at the at the heart of it.”
**Theme:** Societal pressure to act on the topic of childhood obesity at the time spurred adoption of the policy Outer Setting ⇒ *Societal Pressure*	*Role: Other implementation support—Community advisor:* “But in terms of then the state, so I think, you know, the folks that were at the state at the time were really interested in surveillance. They wanted to be on top of what was going on in terms of increasing levels of overweight and obesity and the disparities and watch the disparity be really cognizant of what was going on to our population and the age of children. I mean, it was it was younger and younger children were being impacted by, you know, moving categories of overweight. It was pretty you know, in those days…it was kind of scary to watch…that change in the body weight of our children and so at its root was really the surveillance piece.”
	*Role: High-level leader—MDPH:* “So it was like you added on each piece as a new health issue arrived and obesity was a big one. That was a big 2007, 2008 and we had a data system demonstrating the increase in type one diabetes, type two diabetes. We certainly had asthma. It's all related…So our data system crossed, I think we had 36 health conditions. We were tracking on 1.2 million students, I would say. So obesity was a big one because we you know, we could prevent a lot of chronic illnesses. And so we were absolutely devoted to doing something about this.”
**Aim 2: To identify contextual factors influencing the removal of the BMI report card policy**	
**Theme:** Reported poor design of the policy—including a perceived lack of involvement of key stakeholders in planning—led to inconsistent implementation and overall dissatisfaction, which ultimately enabled de-implementation Innovation ⇒ *Design*	*Role: Innovation deliverer—School nurse:* “I don't know if they ever consulted school nurses to begin with to find out whether it should have been done. They talk to everybody but school nurses most of the time. I can't tell you how many conversations I've had with DESE [Department of Elementary and Secondary Education], that or a state senator or a representative at some point in the conversation it was, ‘Well, we never thought to talk to the school nurses.’ Why not? Where are the people who are there looking at the kids every single day? We're the ones who have to implement these protocols you put together. Why wouldn't you think to start with us and say, do you recognize a need here? No. So I guess that would be the only thing. And I don't know that they didn't, but I don't know that they did. And their track record is not very good for checking with us first.”
	*Role: High-level leader—MDPH:* “I mean, the level of variation of the implementation of the original reg we heard about anecdotally and meaning not just from parents but also from nursing staff about how things were happening, what they had the bandwidth to do. Like there were requirements for confidentiality and privacy, but we would hear stories both from nurses and from kids and their parents that not really, no that wasn't exactly done that way. Again, I have big, I have pictures in my head of stories that we heard about, about kids being lined up and, like, going behind a curtain together, like with the numbers read out. And it's not private. It's ‘pretend’ private. And I could go anyway. So yeah, there was, whereas in other places tons of resources done very differently. So there was variation.”
	*Role: High-level leader—MDPH:* “But my memory of this is that schools also were there was inconsistency in how they were framing it all, framing the sharing of…information with parents…So theoretically, good framing could minimize stigma, could get parents invested in reducing BMI efforts and in thinking about strategies at home. Like that was the whole idea behind this, right, is that parents, you've got a role in this. Your kids can be healthier.”
	*Role: High-level leader—MDPH:* “So even though parents were hesitant to get these BMI report cards, and they were, oh, one of the other problems almost forgot a major problem with the reporting that was occurring initially was that these report cards, as we call them, were being sent home with kids on the school bus or in their backpacks or whatever. And often other kids would get a hold of them. And there was, as you can imagine, major bullying as a result because kids learned how to identify abnormal those that were overweight and those that were being labeled obese. And so that became a huge public nightmare, public relations nightmare for the school district, but also for us. And so that that became problematic.”
**Theme:** The interplay between mass media, societal pressure, and internal pressure and politics was critical to de-implementation Outer Setting ⇒ *Societal Pressure* Inner Setting ⇒ *Tension for Change*	*Role: Other implementation support—Community advisor:* “I do remember this seemed to be a media topic of interest, like the media would like to report on this kind of stuff. And I remember either newspaper or TV report sort of, with parents sort of feeling like this was not information they needed to hear from the schools. That it was an overreach on the part of DPH.”
	*Role: Innovation deliverer—School nurse:* “But when it was being eliminated…I do remember letters to the editor. I remember discussions on the news, interviews with medical professionals as well as nurses, school nurses and parents. So, yeah, I do remember that by the time it was getting eliminated, there were a lot of people who were very upset over it.”
	*Role: Innovation deliverer—School nurse:* “I definitely remember receiving phone calls from parents, you know, but I was really fortunate because when I explained the purpose of this and what was happening, I have to say that I really didn't have any continued backlash about it.”
	*Role: High-level leader—MDPH:* “We didn't get complaints, a lot of complaints from parents.”
	*Role: High-level leader—MDPH:* “So, you know, this department of public health that was rocked by scandal, two different scandals…And so…that had happened, then losing stable leadership, and then Saturday Night Live making fun of the school nutrition regs. We have a brand new commissioner and are trying to make decisions about what's best for children. It was very complex, heightened, heated, terrible.”
	*Role: High-level leader—MDPH:* “And so the Department of Public Health was in this kind of free fall where the public had questions…So we were in a bad light in the public with the legislature and with the governor.”
	*Role: High-level leader—MDPH:* “The timing thing, too. There was emerging evidence in that a study had just been published, which I don't remember any of the details about it now suggesting that the like nutrition regs and the BMI reg in and of themselves weren't the problem, but this parental notification was not evidence based. And third, in complete transparency, the political pressure made it necessary…to move much faster on addressing that regulatory problem than I think would have happened otherwise. So in other words, I think I do think without the kind of political and communications problem, like health communication problem that was coming because of the how much publicity there was about the letter, like ‘fat letters,’ it meant that he [the governor] had to work faster than it normally would in changing a regulation because the evidence was still emerging that it was not good to do.”
	*Role: High-level leader—MDPH:* “Through some other evidence-based research that was being done at the time*–*I know specifically a California study was being cited*–* it was determined that we needed to change the effort from an individual child report to a more community wide report, so a more school-based report versus individual child. So that's when that change started to come about towards the probably the end of 2012. I'll say that we started to look at the data that we had and the fact that it really wasn't working that well in the individual child…But in the process of all of this, it started to come out that parents weren't happy with the reporting structure.”
**Theme:** Perceptions that BMI reporting to parents was not necessary and not appropriate for schools to be doing contributed to dissatisfaction among some participants Inner Setting ⇒ *Mission Alignment*	*Role: Innovation deliverer—School nurse:* “[I] don’t know that other school staff are concerned about anything, but I do know that some of the nursing staff were concerned about it being their responsibility and they weren’t comfortable with that. So the reasons that I have already talked about that the feeling was that this was something that should be done by a primary care provider in that type of setting so that that and that students were already having physicals, so they were already having their weights done and if they needed a referral to PCP was already doing that.”
	*Role: High-level leader—MDPH:* “I remember approaching pediatricians and they really did not want to do what they pediatricians are reluctant to add anything to their plate when it comes to doing initial screenings.”
	*Role: Other implementation support—Community advisor:* “And particularly in those early days, I mean, the pediatricians were not yet giving this information back to students. And I think that was a lot of the impetus behind the state is that they really wanted a surveillance system.”
**Theme:** Communication breakdown contributed to inconsistent implementation Inner Setting ⇒ *Communication*	*Role: High-level leader—MDPH:* “I wasn't aware if there was any required reporting to the school, like to our school nurse part of the department around what they did, how they distributed the information. All we had was anecdotal and I think that's what all of our folks had.”
	*Role: Other implementation support—Community advisor:* “And I don't believe that we had any formalized reporting structure. So I don't think, for example, on the report card, like there was something that said, you know, if you have any concerns about this information, please contact this number or send us an email. So I don't think there was if there was flak from parents or teachers or school nurses or whatever, I don't think we had a well-organized reporting structure. So [it] would have all been at the local level that people if parents were upset, it would be a school committee or school board kind of complaint. So I don't think there was anything that was designed to capture that information. So I don't think we were necessarily in a good place to be systematic in understanding whether there were negative consequences of any importance or prevalent or common commonly experience.”
	*Role: Other implementation support—Community advisor:* “And then parents got this letter that just said, ‘Your kid’s over, by the BMI chart, your kid is overweight.’ [It] didn't take into account, I don't think the letter said anything about well, what this means is if your child is athletic, they may not. And we didn't do any real great education around it. And so it was, it landed with a thud, essentially.”
**Theme:** Uptake of and access to appropriate training, as well as reported gaps in the content of available training, contributed to inconsistent implementation and discomfort Inner Setting ⇒ *Access to Knowledge*	*Role: High-level leader—MDPH:* “We hired a staff member whose job was 40 h a week was to go around to school districts and train school nurses and the others that were going to be involved. As I said, they often had the physical education teachers or others involved in their screenings. We went around and she literally went to individual schools and districts and did extensive training on a daily basis for, oh, 6 to 8, maybe 9 months. That's all she did. And so and we reached…I think we did the entire state. I don't think anybody said no to offering this. Obviously, it was a free training and we went in and brought the equipment in.”
	*Role: Other implementation support—Community advisor:* “I was involved in a survey that we did with school nurses, and they [school nurses/staff] weren't prepared whether we did any training or not. I don't really remember, honestly, but they were not prepared. And nobody, I don't think, still is prepared to figure out how to talk about overweight in a way that is, I mean, it's a stigmatizing condition that we have as a society…have allowed to stigmatize too, in my opinion. But you know, they were not prepared and they hated it, and because they were on the front line having to talk to parents and deal with parents.”
	*Role: Innovation deliverer—School nurse:* “And I remember them [MDPH] offering the trainings and we had regional nurse leader meetings as well. And I'm pretty sure that we…reviewed the change in the in the regulation at the regional meetings and they did offer trainings.”
**Aim 3: To understand the acceptability and feasibility for policy de-implementation**	
**Theme:** The acceptability of de-implementation was not universal Innovation ⇒ *Design* Outer Setting ⇒ *Local Attitudes* Inner Setting ⇒ *Relative Priority*	*Role: Innovation deliverer—School nurse: “*I think like some nurses were just glad to not do it and I'm sure there were other nurses like myself. I was, I would say, indifferent or ambivalent is the right word. But it wasn't like, ‘Oh, we really need to do this kind of a thing.’ It was just kind of like, ‘Oh, okay, we're not going to do that anymore.’
	*Role: Innovation deliverer—School nurse: “*I didn't think it was an effective tool to begin with. So when they said it was being de-implemented, I was very good with that…All it's providing is some statistics that I'm not even sure of the value of the statistics, because we're not taking into account the entire person and what's going on. And so I think for me personally, it was a relief to not have to send that home anymore.”
	*Role: Other implementation support—Community advisor: “*I don't see a downside from it being discontinued except…our heads went back into the sand, and there was now… no information on the health of a child and the dangers of overweight/obesity unless they have some nutrition education in their schools.”
	*Role: High-level leader—MDPH: “*And I think parents’ priorities and perspectives all changed that time because everybody was so focused on these SBIRT [Screening, Brief Intervention, and Referral to Treatment] screening…BMI and postural kind of got downplayed in priorities not only among schools, but among parents. And so we rarely had any complaints from that, but they were still being done.”
	*Role: High-level leader—MDPH:* “But parents became very upset that they weren't getting this information and that their children were coming home and told that they were being weighed. But none of the results were being told to parents…And it became a huge nightmare for the school districts…And we gave that feedback to the department in 2013, 2014 that this wasn't working this procedural way that they had determined was not working.”
	*Role: High-level leader—MDPH:* “Yeah, I think I think it went pretty easily…There are portals for all parents and students to go into. So the letter was in, I believe in the portal and they could access it if they wanted it and not if they didn't want it…Each school, I think, made a different decision. Some of them didn't post them at all—the parent had to request it and had them post it in a place that they could get access to…So I think that each school had hearings about what the tenor of their community around, whether they wanted to know it or not. I would say most they just easily got posted on the portal.”
**Theme:** Perspectives of how childhood obesity can be prevented have evolved since the time of the MA BMI report card policy Innovation ⇒ *Design*	*Role: High-level leader—MDPH: “*Well, I do want to say I want to make it clear from my perspective that the original initiative in 2008, as well intentioned as it was and based upon the research that we had at that time, was flawed in that what we learned between 2008 and 2013 is that it does have to be a community-wide approach. You cannot approach obesity in public health from an individual perspective. That will never work. And I think that was a key lesson learned here, that in the transition between the two got lost, that this is what we are trying to do. Even though school nurses, as I mentioned, had been doing a lot of physical activity initiatives, something to promote that active living and certainly healthy eating during this time. What the message that was getting lost is this takes all of us to if we're going to reduce obesity among our school age children. And that's I'm sorry that that message got lost because that's an important message. It takes a community, a village to fix this.”
	*Role: Other implementation support—Community advisor*: “I mean, we all knew it was like such a bigger problem than what a parent was going to solve overnight or that a kid could take on. But that awareness is part of any kind of strategy and bringing that level of awareness. I'll tell you what it got. It got on people's radars where it wasn't before and the report cards probably weren't particularly impactful at an individual level, but they did raise awareness, I would say, at a community level. Not all of it maybe been positive, but it did bring that attention really forward.”
**Theme**: Not all components of the policy were perceived as needing to be de-implemented Innovation ⇒ *Design* ⇒ *Relative Advantage*	*Role: High-level leader—MDPH:* “Because for surveillance…as well as trying to understand what was working in efforts to address obesity, and so it was…important to maintain the BMI measurement. And so there was this dual goal and of wanting to keep something that was an important part of the regulation and having to move very quickly on something that it was not clear was an important part of the regulation. In fact, the study that was literally published as the regulation was being revisited—they came out was that this piece of the reg was not evidence based.”
	*Role: Other implementation support—Community advisor:* “It's just not worth the…it's not so clear that it has like this amazing impact…and we can continue with the surveillance.”

### Findings for Aim 1: To explore the reasons for adoption of the BMI report card policy

We identified two themes for adoption of the BMI report card policy. The first theme was that the evidence base was not the primary motivator for adoption—instead, the fact that the BMI report cards came from trusted sources mattered more *(Innovation domain: Evidence-base, Source).* One MDPH respondent noted, “But an interesting factoid in terms of how policies are made, it's not really when we always say follow the science.” Instead, participants expressed that seeing a pilot program with vocal champions in the nearby Cambridge Public Schools, and policies in other states, convinced public health leaders to adopt the policy. There was also a sense of trust within MDPH and among the MA Public Health Council of having good intentions and selecting the best available strategies for childhood obesity (see Table 1).

The second theme was that societal pressure to act on the topic of childhood obesity at the time spurred adoption of the policy *(Outer Setting domain: Societal Pressure)*. Participants described how childhood obesity had become an urgent topic, and thus, MDPH felt pressure to implement interventions, even if strong evidence for their effectiveness was not available yet (see Table 1). One MDPH respondent said, “[It] was a full-fledged priority. That’s what it was. There was a priority at the department at the time to respond to the growing epidemic,” and another noted, “We also knew that we didn’t know enough about the obesity epidemic and we didn’t know about effective, evidence-based strategies for the epidemic at the time.”

### Findings for Aim 2: To identify contextual factors influencing the removal of the BMI report card policy

We identified five themes related to factors influencing de-implementation of the BMI report card policy. One theme was that the reported poor design of the policy—including a perceived lack of involvement of key stakeholders in planning—led to inconsistent implementation and overall dissatisfaction which ultimately enabled de-implementation *(Innovation domain: Design)*. Reported minimal involvement of school nurses in the planning of the implementation process—despite being responsible for program delivery—may have led to inconsistencies in how schools conducted BMI screening and/or reporting. An MDPH participant commented, “We had a lot of difficulties in maintaining consistency and standards.” Under representation of school nurses in program design may have also contributed to dissatisfaction with the policy (see Table 1).

Another factor identified was that the interplay between mass media, societal pressure, and internal pressure and politics was critical to de-implementation *(Outer Setting domain: Societal Pressure; Inner Setting domain: Tension for Change).* Just as societal pressure influenced policy adoption, external pressures to reconsider the policy were major catalysts in its reversal. Some MDPH leaders described learning BMI report cards may not be effective through internal review of the surveillance data and a 2011 study [16]; however, this did not prompt de-implementation. Instead, participants recalled how BMI report cards were mocked as “fat letters” during an episode of the television show *Saturday Night Live*, and that this accelerated de-implementation. One MDPH staff noted, “And so it hit the news waves. It was on CNN. Saturday Night Live did a little skit about the ‘fat letter’. And all of a sudden I’m in the governor’s office: ‘get rid of BMI’.” Additional news stories featuring upset parents made the governor work quickly to de-implement it (see Table 2 for media examples). Parental concerns raised in the media mirrored the occasional complaints reported by school nurses though MDPH staff voiced mixed perspectives on the volume of parent complaints received since implementation (see Table 1). This societal pressure to end the policy coincided with a challenging time at MDPH, which had recently experienced changes in leadership and staffing following the department’s involvement in several incidents of misconduct unrelated to the BMI report cards. MDPH, in an effort to re-gain favor with the public, felt a responsibility to respond to the public outcry over BMI report cards. One MDPH staff noted, “We have a brand-new commissioner and are trying to make decisions about what's best for children. It was very complex, heightened, heated, terrible.”

**Table 2:** Examples of media coverage related to the MA BMI Report Card Policy mentioned in interviews and from authors’ search (2009–2013)

Title	Source	Date
This is the letter you get if Massachusetts thinks your kid is too fat	BuzzFeed	2/26/2013
‘Fat letters’ sent home to students spark controversy In Massachusetts	HuffPost	2/28/2013
‘Fat letters’ sent to parents of obese Massachusetts students	ABC News	2/28/2013
‘Fat letters’ sent to parents no laughing natter	Boston Magazine	3/1/2013
Weekend Update with Seth Meyers	Saturday Night Live	3/2/2013
BMI measuring in schools proves weighty issue	Chicago Tribune	5/17/2013
Families protest so-called fat letters	Yahoo News	9/10/2013
Parents still outraged over ‘fat letters’	Boston Magazine	9/12/2013
‘Fat letters’ from schools to parents are wrong	CNN	9/24/2013
Massachusetts scraps controversial student obesity letters	Boston.com	10/16/2013
Rethinking public school ‘fat letters’ for students	Time	10/17/2013
Massachusetts schools to stop sending ‘fat letters’	US News	10/17/2013
‘Fat letters’ to be eliminated in Massachusetts; Public schools will no longer tell parents whether their kids are obese	Medical Daily	10/17/2013

Perceptions that BMI reporting to parents was not necessary and not appropriate for schools to be doing contributed to dissatisfaction among some participants* (Inner Setting domain: Mission Alignment)*. Specifically, there was a perceived mismatch between the role of schools versus pediatricians’ offices in collecting and discussing BMI with families. Some respondents believed addressing BMI with parents was best suited for pediatricians and/or that pediatricians were already screening BMI. One school nurse said, “I don’t see any reason why schools need to be involved in this issue…[It’s] doubling up on information that we already have.” However, other respondents felt that pediatricians were *not* actually discussing BMI with families, and thus this intervention was needed (see Table 1).

A fourth catalyst for de-implementation was communication breakdown contributed to inconsistent implementation *(Inner Setting domain: Communication).* Respondents expressed disconnects between policymakers, practitioners, and families impacted by the policy. Some involved with developing the policy procedures reported having minimal opportunity for feedback from those affected by the policy. One such community advisor said, “I don’t think we had a well-organized reporting structure…So I don’t think we were necessarily in a good place to be systematic in understanding whether there were negative consequences.” Similarly, there were miscommunications about the intention of the report cards between those who designed or delivered the intervention and the families (see Table 1). One MDPH participant remarked,

And one of the things we had to keep stressing…that this is simply a screening, it is not a diagnostic tool… So it was kind of like you were calling them obese, but not really. So there's a lot of confusion. And school nurses were confused by it, too.

A fifth catalyst was that uptake of and access to appropriate training, as well as reported gaps in the content of available training, contributed to inconsistent implementation and discomfort *(Inner Setting domain: Access to Knowledge)* (see Table 1). While one MDPH leader reported providing exhaustive training, a school nurse said, “I know I never received any training.” Another public health practitioner involved in supporting implementation characterized the training as not helping nurses address the sensitive nature of reporting children’s BMI, saying, “We didn’t provide the appropriate training for school nurses, the appropriate sensitivity training, culturally appropriate training, the whole gamut…it was just height, weight.”

### Findings for Aim 3: To understand the acceptability and feasibility of policy de-implementation

We identified two themes related to acceptability. The first theme is that acceptability of de-implementation was not universal (*Innovation domain: Design; Outer Setting domain: Local Attitudes; Inner Setting domain: Relative Priority).* One school nurse reflected, “I think as a collective group, we [school nurses] felt relieved that we…no longer have to do that,” whereas others disagreed (see Table 1). In the school year following de-implementation, one MDPH participant recalled a surge in complaints about why schools were collecting student BMI, but not reporting it to parents/guardians: “But parents became very upset that they weren’t getting this information and that their children were coming home and told that they were being weighed.”

The second theme for acceptability is that when reflecting on de-implementation, some within the MDPH, the Public Health Council, and community advisory groups remarked how perspectives of how childhood obesity can be prevented have changed since the time of the MA BMI report card policy *(Innovation domain: Design).* Respondents described how the public health field has evolved to consider the social, structural, and economic determinants of health when addressing obesity rather than focusing solely on individual behavior change (see Table 1). A member of the MA Public Health Council said:

The idea of approaching a problem like obesity with such an individual focused intervention just really flies in the face of everything we know about the structural issues that are responsible for obesity. And so it seems very obvious to me now that this is like using a hammer on I don’t know, something that’s not a nail.

We identified one theme for the feasibility of policy de-implementation in that not all components of the policy were perceived as needing to be de-implemented (*Innovation: Design, Relative Advantage).* There was agreement that it was favorable to retain the BMI surveillance component to allow for public health planning and evaluation. One MDPH participant said,

You know, I think surveillance is a very important thing. And I think that if we were to lose the ability to track the impact of the whole range of prevention and health promotion strategies that were happening, both in schools and outside of schools through this through BMI measurement and reporting would have been not good.

By retaining BMI measurement and reporting to MDPH only, leaders were able to optimize acceptability, particularly within the MDPH, and feasibility, lessening the burden on individual schools (see Table 1).

## Discussion

Our qualitative study of the MA BMI report card policy identified multiple themes about why the policy was implemented in 2009 and what catalyzed its de-implementation in 2013. Our findings align primarily with one criterion for de-implementation proposed by McKay et al.: the intervention is found to be ineffective or harmful [1]. However, several factors may matter *more* than the evidence of an intervention’s effectiveness for implementation and de-implementation, especially when there is tension between the Inner and Outer Setting domains. It appeared that societal pressure for public health leaders to quickly address childhood obesity, before there was ample research, meant that leaders were spurred to implement a policy without strong evidence behind it. Yet the policy was still not de-implemented when evidence had emerged at that time suggesting it was not effective [16] or that it could exacerbate body image concerns [29] and unhealthy weight management behaviors [30]. Instead, societal pressure again played a pivotal role when state leadership received national attention and political pressure to de-implement increased. MDPH’s concerns about reputation at the time and the ongoing reorganization of staff added a layer of internal pressure for de-implementation. Initial problems with implementation, including perceptions of limited engagement of school nurses in program planning, miscommunication, training gaps, and a sense of mission misalignment, contributed to tepid support, which may have also allowed it to be more easily de-implemented.

Consistent with Prusaczyk’s hypothesis that factors influencing implementation may differ from those for de-implementation [3], our findings offer an applied example of such differences for a public health policy. Innovation design, internal pressure in the Inner Setting, and societal pressure in the Outer Setting, and their interaction, were highly influential for prompting policy adoption and de-implementation. However, pressure appeared to be much higher across both levels of the Inner Setting (MDPH and schools) and the Outer Setting for de-implementation. We also found differences in the acceptability and feasibility of de-implementation for the *practice* of BMI report cards versus the *process* of de-implementation [3]. Opinions were mixed on the decision to de-implement the policy based on considerations for the Innovation, Inner Setting, and Outer Setting domains. The process for de-implementation, however, appeared to be straightforward, although some felt there could have been more efforts to first test the model of BMI screening only. A final consistency with Prusaczyk’s conceptual paper [3] is that we observed variation in respondent perspectives within and between roles and by policy implementation and de-implementation which could in theory contribute to incomplete de-implementation. In this case, however, it does appear that de-implementation was successful, especially since school nurses expressed a sense of relief for no longer having to issue BMI report cards. However, our study team did encounter several instances in our formative research where it was unclear from online materials or communications with school professionals about whether schools were still required to implement the BMI report card component. Replication of information from outdated websites, especially when only part of the original intervention is de-implemented, could present challenges to complete de-implementation of public health policies.

Weno et al. developed a taxonomy of strategies for de-implementation of public health programs [31]. The first strategy is to use evaluation data for decision-making. While some participants noted being aware of new evidence and state data showing BMI report cards were not decreasing childhood obesity, this was not a primary catalyst for de-implementation. A second strategy, to consider if any program components can be saved, did take place; MDPH maintained the requirement to measure BMI at school and report aggregate data to MDPH while removing report cards. A third strategy, to transparently communicate and discuss program adjustments, was not fully utilized. While the policy amendment was presented at an MA Public Health Council meeting prior to de-implementation, some participants believed the revised policy should have been piloted before de-implementation occurred statewide to test acceptability and avoid unintended consequences. The additional strategies suggested by Weno et al.—respect partner relationships and communicate effectively—do not appear to have been primary strategies used for de-implementation. This is unsurprising since this de-implementation framework did not exist in 2013; however, this raises a larger question of what types of processes decision-makers use to de-implement public health interventions that have fallen out of favor and whether there are ideal processes for de-implementation. Our results suggest, at least in considering public health *policies* specifically, it may be useful to apply Kingdon’s policy window model, which posits the need for alignment of three streams for policies to be implemented: problem, policy, and politics [32]. Our findings indicate this window of opportunity may also be present in policy de-implementation, particularly when de-implementation occurs in response to external factors rather than being planned.

As research evolves and commonly used interventions are found to be ineffective, the question of how to de-implement is important. Public health practitioners, in response to urgent public health problems, may need to act before evidence for action is available. Thus, practices may become incorporated into an institution’s work and be difficult to change. Our study suggests there is not a well-defined process for de-implementing interventions driven by policy change; rather, the de-implementation of this policy depended largely on chance and political processes. Future research should identify effective de-implementation strategies for interventions that can be easily used by public health leaders. The de-implementation of ineffective interventions has the added benefit of freeing up resources for effective ones, a critical issue considering the limited availability of public health dollars. Surprisingly, costs were not reported as a consideration for de-implementation in this study. BMI report cards are relatively inexpensive per student [10, 33], but the labor time required for school nurses to implement BMI report cards over time is not insignificant [10] which was echoed in some school nurse interviews.

This study had several limitations. Our sample size was small and may not have represented all perspectives on the implementation and de-implementation of the MA BMI report card policy. Our focus on state-level processes meant we could not incorporate the perspectives of those who were most directly affected by the intervention (i.e., students and parents/guardians). However, we did include perspectives of an array of individuals involved in policy adoption, implementation, and de-implementation. The results of our study may be limited by the time gap between the implementation of the policy in 2009, de-implementation in 2013, and our interviews in 2022. While we verified key events with a review of historical documents and reminded participants of the timeline for implementation and de-implementation as part of the interview script, participants were not asked to review our study results which could have strengthened the validity of our findings. Additionally, it is possible that currently held beliefs and experiences could have biased participant recall; however, we included an interview question that asked explicitly for interviewees to contrast their current perspectives with those from the time of the MA BMI report card policy.

## Conclusions

Societal pressure, political pressure, and problems with initial design and implementation were key catalysts for the de-implementation of the MA BMI report card policy. Public health research should further focus on developing strategic processes to de-implement ineffective policies and interventions that adequately address the drivers of policy de-implementation.

## Supplementary Information

**Additional file 1.** Standards for ReportingQualitative Research (SRQR).

**Additional file 2:** **Appendix. **InterviewGuide.

## Data Availability

Data sharing is not applicable to this article as no datasets were generated or analyzed during the current study.

## Abbreviations

BMI: Body mass index
MA: Massachusetts
MDPH: Massachusetts Department of Public Health
CFIR: Consolidated Framework for Implementation Research
