# Supplementary material for: De-implementing public health policies: a qualitative study of the process of implementing and then removing body mass index (BMI) report cards in Massachusetts public schools
Source: Implement Sci Commun. 2023 Jun 9;4:63. doi: 10.1186/s43058-023-00443-1 (PMC10251595; doi:10.1186/s43058-023-00443-1)
Supplement: Supplementary file 2 — Additional file 2: Appendix. InterviewGuide. [file 43058_2023_443_MOESM2_ESM.docx]

**Appendix. Interview Guide**

**I. BACKGROUND**

1. First, to help me understand who was involved MA BMI report card policy, will you please describe where you worked and what your roles was when the policy was passed in 2009?

*PROBES:*

- What division/department did you work in?
- What was your title within your organization?
- Who did you report to?
- How were you connected to the policy? How did you get involved?

1. Were you still in this role when the policy was de-implemented in 2013? If yes, move to next question. If no, walk through probes below:

*PROBES:*

- What division/department did you work in?
- What was your title within your organization?
- Who did you report to?
- How were you connected to the policy? How did you get involved?

**II. POLICY ADOPTION & IMPLEMENTATION**

In 2009, MA BMI report card policy was adopted by the MA Public Health Council. I’d like to start by discussing what that experience was like.

1. How were you involved in this policy adoption and implementation? What do you remember about the experience?
2. First, what type of evidence was considered when putting this policy is place?

- *Probe for any specific research (e.g., Fitnessgram study) or practice experience of other states.*

1. Very broadly, can you describe the major factors (positive or negative) that you think influenced adoption of this policy?

*PROBES:*

- What about the characteristics of the individuals who supported adoption of the program (e.g., background and skillset, role and experience of those involved)?
- What about the climate at MDPH? (e.g., leadership support, available resources & funding, data systems, internal policies and processes, staff buy-in) *(inner setting)*
- How about the influences outside of MDPH (e.g., political climate, Local, state, or national policies; community context; public awareness/demand)?
- Finally, what about the characteristics of the policy itself? (such as the complexity of the policy)
- Also: factors described in meeting minutes (e.g., parent awareness of BMI, healthy habit promotion, connection to primary care, evaluation, experience leading up to adoption)

1. What did you think about the concerns that were raised at the time of adoption (e.g., disordered eating, bullying, weight stigma)?
   - *What were the safeguards that were put in place? To what degree to you think they were effective?*
2. Next, we’d like to get your reflections on the guideline manual development process. Can you describe that process? Who was involved? Want major consideration were made?
   - *Any trainings or TA that were available for it?*
   - *Any changes to screening approach already in place vs. just adding in reporting?*
3. In terms of the implementation process at the school level, what did that look process look like?

*PROBES:*

- *Who was involved with implementation?*
- *When did implementation occur?*
- *Was the policy easy or difficult to implement?*
- *Were there trainings or other assistance available to staff to help deliver?*

1. Very broadly, can you describe the major factors (positive or negative) that you think influenced implementation of this policy?

*PROBES:*

- What about the characteristics of the individuals responsible for implementation of the program (e.g., background and skillset, role and experience of those involved)?
- What about the organizational context of the schools? (e.g., leadership support, available resources & funding, data systems, internal policies and processes, staff buy-in) *(inner setting)*
- How about the influences outside of schools (e.g., political climate, Local, state, or national policies; community context; public awareness/demand)?
- Finally, what about the characteristics of the policy itself? (such as the complexity of the policy)
- Also: anticipated barriers from meeting minutes (e.g., burden to nurses).
- Any changes to screening approach already in place vs. just adding in reporting?

1. What were parent and student reactions to the new policy like?
2. Can you think of any potential unintended negative consequences that were noticed as the policy was put in place? What were they?

- *Probe on decreasing vision and hearing screening, weight stigma, disordered eating*

**II. POLICY DE-IMPLEMENTATION**

In 2013, the MA BMI report card policy ended. I’d like to shift gears to discuss what that experience was like.

1. What did you think about the policy ending?
   - *Probes: Was it a good idea or not?*
   - *Has your perspective changed since 2013?*
2. How were you involved in the process of ending the policy? What do you remember about the experience?
3. What type of evidence was considered that made people want to end the policy?

- *Probe for any specific research or practice experience of other states.*

1. Very broadly, can you describe the major factors (positive or negative) that you think influenced ending this policy?

*PROBES:*

- What about the characteristics of the individuals responsible for implementation of the program (e.g., background and skillset, role and experience of those involved)?
- What about the organizational context of the schools? (e.g., leadership support, available resources & funding, data systems, internal policies and processes, staff buy-in) *(inner setting)*
- How about the influences outside of schools (e.g., political climate, Local, state, or national policies; community context; public awareness/demand)?
- Finally, what about the characteristics of the policy itself? (such as the complexity of the policy)
- Also: anticipated barriers from meeting minutes (e.g., burden to nurses).

1. In terms of the process for ending the policy at schools, what did that look process look like?
   - *Were there any communications from MDPH to the schools?*
   - *Was there school level variation in how schools ended it?*
   - *Did some keep it in place?*
2. What were parent and student reactions to the policy ending?

**CLOSING**

1. Stepping back almost decade later, do you think things should have gone differently? What would you have changed? Adoption altogether? Implementation Process? De-implementation?
2. Do you have any suggestions for other states that currently have or are considering implementing policies mandating BMI report cards? What would you tell them about your experience and what you’ve learned?
